# Ribosomal DNA clusters and telomeric (TTAGG)n repeats in blue butterflies (Lepidoptera, Lycaenidae) with low and high chromosome numbers

**DOI:** 10.3897/CompCytogen.v9i2.4715

**Published:** 2015-05-11

**Authors:** Alisa O. Vershinina, Boris A. Anokhin, Vladimir A. Lukhtanov

**Affiliations:** 1 Zoological Institute, Russian Academy of Sciences, Universitetskaya emb. 1, St. Petersburg 199034, Russia; 2 Department of Entomology, St. Petersburg State University, Universitetskaya emb. 7/9, St. Petersburg 199034, Russia

**Keywords:** Lycanidae, ribosomal DNA, chromosome, taxonomy, karyotype evolution, telomeres

## Abstract

Ribosomal DNA clusters and telomeric repeats are important parts of eukaryotic genome. However, little is known about their organization and localization in karyotypes of organisms with holocentric chromosomes. Here we present first cytogenetic study of these molecular structures in seven blue butterflies of the genus *Polyommatus* Latreille, 1804 with low and high chromosome numbers (from n=10 to n=ca.108) using fluorescence *in situ* hybridization (FISH) with 18S rDNA and (TTAGG)*_n_* telomeric probes. FISH with the 18S rDNA probe showed the presence of two different variants of the location of major rDNA clusters in *Polyommatus* species: with one or two rDNA-carrying chromosomes in haploid karyotype. We discuss evolutionary trends and possible mechanisms of changes in the number of ribosomal clusters. We also demonstrate that *Polyommatus* species have the classical insect (TTAGG)*_n_* telomere organization. This chromosome end protection mechanism probably originated *de novo* in small chromosomes that evolved via fragmentations.

## Introduction

Most studied butterfly families and genera share the modal chromosome number of n=30 or n=31 (Robinson 1971) and this, most likely ancestral chromosome number is maintained in the Lepidoptera karyotype evolution (Suomalainen 1979, Lukhtanov 2000, 2014). The vast majority of Lepidoptera species have also similar karyotype structure with all the chromosomes being of a similar size or forming gradually increasing size series (Lukhtanov and Dantchenko 2002). The uniformity of karyotypes does not imply that chromosome rearrangements were not involved in genome evolution in butterflies and moths. Numerous inter- or intrachromosomal rearrangements such as translocations and inversions, can contribute to karyotype evolution without significant changes in chromosome number and size. However, detecting these rearrangements is difficult due to several specific properties of Lepidoptera karyotype. *Lepidoptera* and their sister group, caddisflies (Trichoptera), have holocentric chromosomes, i.e. chromosomes without localized centromeres (Wolf et al. 1997), and this makes impossible using the centromere as a marker. Attempts to use differential banding techniques have appeared but were inefficient (Guerra et al. 2010).

These are the reasons explaining why the karyotype evolution is still poorly understood in Lepidoptera, though some data regarding karyotype organization and genome rearrangements are present for *Bombyx mori* (Linnaeus, 1758) (Yoshido et al. 2005), *Heliconius melpomene* (Linnaeus, 1758) (Pringle et al. 2007), *Bicyclus anynana* (Butler, 1879) (Van’t Hof et al. 2008), *Samia cynthia* (Drury, 1773) (Yoshido et al. 2011), *Biston betularia* (Van’t Hof et al. 2013), and *Melitaea cinxia* (Linnaeus, 1758) (Ahola et al. 2014).

A molecular hybridization technique, such as fluorescence *in situ* hybridization (FISH), is a very useful method for studying molecular organization of chromatin and for tracing individual chromosomes in different species (Pinkel et al. 1986). FISH markers, specifically rDNA clusters, were proposed for some insects (Colomba et al. 2000, Grozeva et al. 2010, 2011, Gokhman et al. 2014, Panzera et al. 2012, 2014) including butterflies (Nguyen et al. 2010). Ribosomal gene clusters consist of rDNA arrays and as a part of nucleolus organizer regions (NORs) form the nucleolus during interphase (Scheer and Hock 1999).

The sparse data available have contributed to generalizations about the pattern and mode of the major rDNA cluster evolution in Lepidoptera. According to Nguyen et al. (2010) rDNA distribution in Lepidoptera is a result of dynamic evolution with the exception of Noctuoidea, which showed the static rDNA pattern. In a compilation with previous data they also hypothesize multiplication of rDNA clusters as a trend in the Lepidoptera karyotype evolution. Using specimens with dramatically different high and low chromosomal numbers we aim to examine the association between karyotype and rDNA cluster number. Thus, as a model we have chosen blue butterflies of the subgenus *Agrodiaetus* Hübner, 1822, which includes about 130 described species within the genus *Polyommatus* Latreille, 1804 (Lepidoptera, Lycaenidae) (Talavera et al. 2013a). This subgenus exhibits a wide diversity of karyotypes, with haploid chromosome numbers of different species ranging from 10 to 134 (Lukhtanov et al. 2005, 2014, Vershinina and Lukhtanov 2010). The variability is not associated with polyploidy and is caused by multiple chromosome fusions and fissions (Kandul et al. 2007). We investigated distribution of ribosomal clusters in karyotypes by mapping 18S ribosomal DNA probe on chromosomes of Polyommatus (Agrodiaetus) caeruleus (Staudinger, 1871), Polyommatus (Agrodiaetus) hamadanensis (de Lesse, 1959), Polyommatus (Agrodiaetus) karindus (Riley, 1921), Polyommatus (Agrodiaetus) morgani (Le Cerf, 1909), Polyommatus (Agrodiaetus) peilei (Bethune-Baker, 1921), Polyommatus (Agrodiaetus) pfeifferi (Brandt, 1938) and Polyommatus (Agrodiaetus) sennanensis (de Lesse, 1959) which are drastically different in their chromosome numbers (from n=10 to n=108).

Additionally, we analyzed molecular organization of telomeric repeats in *Polyommatus* (subgenus *Agrodiaetus*). In animals there are three main types of telomeric tandem repeats: TTAGGG, TTAGGC, and TTAGG. The TTAGGG motif is probably ancestral for all Metazoa and has been found in all multicellular animals, except round worms and arthropods (Traut et al. 2007). TTAGGC repeats are specific for nematodes (Wicky et al. 1996), whereas the TTAGG motif prevails in most arthropod groups providing support for a common origin (Vítková et al. 2005, Lukhtanov and Kuznetsova 2010). The (TTAGG)*_n_* telomeric structure has been demonstrated in several lepidopteran species, such as the silkmoths *Bombyx mori* (Linnaeus, 1758) and *Bombyx mandarina* (Moore, 1872) (Bombicidae, Okazaki et al. 1993, Sahara et al. 1999); saturniid moths *Antheraea pernyi* (Guérin-Méneville, 1855), *Antheraea yamamai* (Guérin-Méneville, 1861) and *Samia cynthia* (Drury, 1773) (Okazaki et al. 1993); the vapourer *Orgyia antiqua* (Linnaeus, 1758) (Lymantriidae, Rego and Marec 2003); the wax moth *Galleria mellonella* (Linnaeus, 1758) and the flour moth *Ephestia kuehniella* (Zeller, 1879) (Pyralidae, Sahara et al. 1999, Rego and Marec 2003). Thus, TTAGG telomeric structure is expected in other butterfly and moth families. However, several exceptions from the (TTAGG)*_n_* motif are known for insects (for additional information see Frydrychová et al. 2004, Lukhtanov and Kuznetsova 2010, Kuznetsova et al. 2011; Gokhman et al. 2014). Exceptions in the telomere structure occur at different taxonomic levels, not only at the level of order but also on the level of infraorder in Heteroptera (Kuznetsova et al. 2012) and Hymenoptera (Gokhman et al. 2014), at the level of family in Curculionidae (Sahara et al. 1999), and even within Curculionidae (Frydrychová and Marec 2002). So far nothing is known about telomeres in Lycaenidae butterflies. Here we study the structure of telomeres in *Polyommatus* (subgenus *Agrodiaetus*) butterflies by using FISH with (TTAGG)*_n_* probes.

## Material and methods

Butterfly species were collected from 2005 to 2011 by V. Lukhtanov, A. Dantchenko and N. Shapoval in Iran (Table 1). Only male adult specimens (from 1 to 5 individuals for each population) were analyzed. In field, gonads were fixed in a solution of absolute alcohol and glacial acetic acid (3:1) and then stored at -4 °C; meiotic chromosomes were obtained from testes, according to the standard protocol for squash preparation (Lukhtanov and Dantchenko 2002, Lukhtanov et al. 2008; Vila et al. 2010). Tissues were prepared in a drop of 45% acetic acid and then fixed on a slide by freezing on a dry ice and following dehydration in a series of ethanol solutions (70-80-96%, 2 minutes each). Prior to DNA hybridization karyotypes were examined by phase contrast microscopy.

**Table 1.** List of *Polyommatus* (subgenus *Agrodiaetus*) populations used in the present study and their haploid chromosome numbers (n) according to original data.

Species	n	Province	Locality	altitude	date
Polyommatus (Agrodiaetus) caeruleus	10	Golestan	Shahkuh	2700–3100 m	2005.VII.22
Polyommatus (Agrodiaetus) hamadanensis	19	Lortestan	Sarvand, 33°22.38'N/ 49°10.25'E	2070 m	2009.VII.22
Polyommatus (Agrodiaetus) hamadanensis	21	Esfahan	Kuhe-Tamandar Mts, 33°12.72'N/ 49°56.43'E	2336 m	2011.VII.16
Polyommatus (Agrodiaetus) karindus	ca.68	Kurdistan	40 km SW Saqqez, 36°04.39'N/ 45°59.06'E	1869 m	2009.VII.29
Polyommatus (Agrodiaetus) morgani	25	Kurdistan	14 km N of Chenareh, 35°42.12'N/ 46°22.35'E	2025 m	2009.VII.28
Polyommatus (Agrodiaetus) peilei	39	-	14 km N of Chenareh, 35°42.127'N/ 46°22.35'E	2025 m	2009.VII.28
Polyommatus (Agrodiaetus) pfeifferi	ca.108	Fars	Barm-i-Firuz Mts, 30°23'N/ 51°56'E	2900 m	2002.VII.19
Polyommatus (Agrodiaetus) sennanensis	27	Qom	Qom-Qamsar, 33°43.80'N/ 51°29.53'E	1862 m	2009.VII.16

18S rDNA and (TTAGG)*_n_* probe preparation and hybridization were carried out as described in Grozeva et al. (2011). In brief, chromosome preparations were treated with 100 µg/ml RNaseA and 5 mg/ml Pepsin solution to remove excess RNA and proteins. Chromosomes were denatured on a slide in hybridization mixture with biotinylated 18S rDNA probe from the genomic DNA of *Pyrrhocoris apterus* and rhodaminated (TTAGG)*_n_* probes with addition of salmon sperm DNA blockage and then hybridized for 42 h. 18S rDNA loci were detected with NeutrAvidin-FITC. Chromosomes were mounted in an antifade medium (ProLong Gold antifade reagent with DAPI, Invitrogen) and covered with a glass coverslip. Images were taken with a Leica DFC 345 FX camera using Leica Application Suite 3.7 software.

### Abbreviations

ca. (circa) approximately.

FISH fluorescence *in situ* hybridization.

MI meiotic metaphase I.

MII meiotic metaphase II.

NOR nucleolus organizer region.

## Results

**Figures 1–8.**
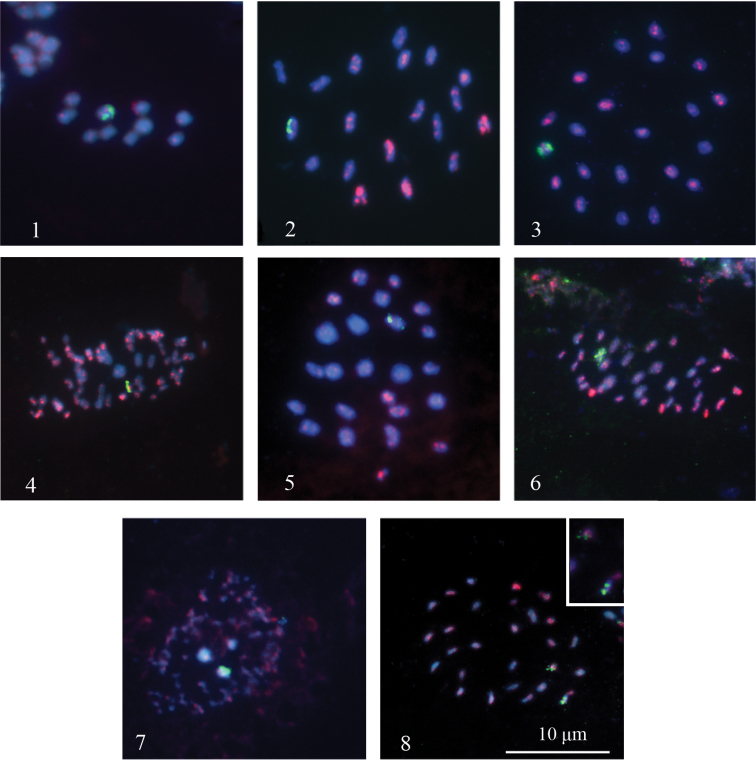
Localization of FISH signals on telomeres (red) and rDNA clusters (green) in squash chromosome preparations of seven species of *Polyommatus* (subgenus *Agrodiaetus*). Chromosomes are counterstained with DAPI. Note telomeric signals of different intensity. **1–7** one 18S rDNA cluster is found **8** two 18S rDNA clusters are found **1** Polyommatus (Agrodiaetus) caeruleus, MII **2–3** Polyommatus (Agrodiaetus) hamadanensis, MI cells from two different populations with different karyotypes (n=19 and n=21 accordingly) **4** Polyommatus (Agrodiaetus) karindus, MII **5** Polyommatus (Agrodiaetus) morgani, MI **6** Polyommatus (Agrodiaetus) peilei, MII **7** Polyommatus (Agrodiaetus) pfeifferi, MII **8** Polyommatus (Agrodiaetus) sennanensis, MII. The inset in the upper right corner shows twice enlarged image of rDNA-carrying chromosomes.

In all karyotypes weak and strong telomeric signals were present (Figs 1–8). The chromosomes of blue butterflies are very small and some of them are at the limit of the resolving power of light microscopy. For this reason, TTAGG signals in some cases could not be distinguished from background noise. Unlike the telomere probes, rDNA probes produced strong signals of different intensity. The chromosomal distribution pattern of telomeric repeats was similar in all seven species, the exact location of telomeres (terminal or interstitial) was impossible to identify since the meiotic chromosomes were extremely contracted. The distribution pattern of 18S rDNA signals varied markedly showing two different variants – with one or two rDNA-carrying bivalents in MI karyotype and, correspondingly, with one or two rDNA-carrying chromosomes in MII karyotype. All chromosome numbers were found to coincide with previously published karyotype data for seven studied species (Lukhtanov et al. 2005, Kandul et al. 2007). In two Polyommatus (Agrodiaetus) hamadanensis populations intraspecific chromosomal polymorphism has been discovered.

Polyommatus (Agrodiaetus) caeruleus had n=10 with one rDNA cluster localized in one of the chromosome pairs (Table 1, Fig. 1). In MII cells this cluster appeared as a combination of two signals, localized on sister chromatids on one of the chromosomes. Weak (TTAGG)*_n_* signals were found in all chromosomes.

In Polyommatus (Agrodiaetus) hamadanensis, the haploid chromosome number of n=19 was found in MI cells of one studied individual from Lorestan province. In the specimens from another population (Esfahan province) the number of n=21 was found in MI cells (Table 1, Figs 2–3). The karyotype had no especially large or small bivalents; all bivalents were nearly equal in size and formed a gradient size series. In both specimens, one rDNA cluster was found. In MI cells this cluster appeared as a combination of two signals, localized on homologous chromosomes of one of the bivalents (Figs 2–3). (TTAGG)*_n_* signals of different intensity were found in all bivalents.

In Polyommatus (Agrodiaetus) karindus, the haploid chromosome number of n= ca.68 was found in MII cells (Table 1, Fig. 4). One rDNA cluster was found on one of the chromosomes. Numerous (TTAGG)*_n_* signals of different intensity were found in all chromosomes. The karyotype had three large chromosomes while the other chromosomes had a relatively equal small size.

In Polyommatus (Agrodiaetus) morgani, the haploid chromosome number of n=25 was found in MI cells of a single individual (Table 1, Fig. 5). One bivalent was found to carry the rDNA site. (TTAGG)*_n_* signals of different intensity were found in all bivalents.

In Polyommatus (Agrodiaetus) peilei, the haploid chromosome number of n=39 was found in MII cells (Table 1, Fig. 6). Strong 18S rDNA signals were observed on one of the chromosomes. (TTAGG)*_n_* signals of different intensity were found in all chromosomes.

In Polyommatus (Agrodiaetus) pfeifferi, the chromosome number was only approximately established and was n=ca.108 (Table 1, Fig. 7). The karyotype had two large, one medium-sized and more than 100 very small chromosomes. In MII cells, a single rDNA cluster was found on one pair of relatively large chromatids. Numerous weak (TTAGG)*_n_* signals were observed in all chromosomes, but their number and localization were difficult to estimate due to the background noise.

In Polyommatus (Agrodiaetus) sennanensis, the haploid chromosome number of n=27 was found in MII cells (Table 1, Fig. 8). In contrast to other studied species, Polyommatus (Agrodiaetus) sennanensis had two distinct rDNA clusters localized on different, non-homologous chromosomes. (TTAGG)*_n_* signals of different intensity were found in all chromosomes.

## Discussion

Previous investigations by Nguyen et. al. (2010) examined ribosomal clusters in 18 species of different taxonomic groups of Lepidoptera. Discussing evolutionary dynamics of rDNA clusters these authors suggest several concepts. One of them implies origin of one interstitial ribosomal cluster on rDNA-bearing chromosome as a result of a fusion between two NOR-bearing chromosomes (Nguyen et. al. 2010). However, their own table (fig. 3 in Nguyen et. al. 2010) shows a different picture: nearly all species with n=31 and haploid chromosome number less than 31 have one (mostly interstitial) rDNA cluster. Our data based on the study of diverse karyotypes in *Polyommatus* (subgenus *Agrodiaetus*) butterflies show a similar pattern. All studied species except for Polyommatus (Agrodiaetus) sennanensis have one rDNA cluster in haploid karyotype regardless of their chromosome number. Therefore, we cannot consider rDNA cluster number reduction via fusion as a common trend in the evolution of Lepidoptera genomes. Rather they tend to preserve the single rDNA cluster, the state which seems to be an ancestral one.

Specifically for blue butterflies (Lycaenidae), Nguyen et al. (2010) suggested the mechanism of rDNA cluster multiplication via chromosome fissions. This hypothesis is based on the facts that *Polyommatus icarus* (Rottemburg, 1775) which has ancestral for Lycaeninae n=23-24 (Robinson 1971) also has a single interstitial NOR whereas *Lysandra bellargus* (Rottemburg, 1775) has two NORs, therewith the chromosome number in *Lysandra bellargus* was increased to n = 45 most likely via fragmentations (Kandul et al. 2007, Talavera et al. 2013b). Thus, Nguyen et al. (2010) hypothesized that the single ancestral NOR-chromosome was likely to split into two fragments resulting in two NOR-chromosomes. According to our data this hypothetical mechanism is, at least, not a general one in Lycaenidae since all the studied species with increased number of small chromosomes (Polyommatus (Agrodiaetus) peilei, n=39; Polyommatus (Agrodiaetus) karindus, n=68 and Polyommatus (Agrodiaetus) pfeifferi, n=ca108) have only one rDNA cluster per haploid genome.

Chromosome fissions lead to strong decrease in size of fragmented chromosomes (Lukhtanov and Dantchenko 2002). However, there is an empirical rule that in Lepidoptera one (or few) chromosome is evolutionary stable and protected from fragmentation; therefore it preserves its ancestral relatively large size whereas the rest of chromosomes are fragmented and small (White 1973). In our results, 18S rDNA probe in Polyommatus (Agrodiaetus) pfeifferi (in which the majority of chromosomes are extremely fragmented) is located on the largest chromosome (Fig. 7) suggesting possible evolutionary stability of rDNA-carrying chromosome.

The third possible mechanism which can change the number of rDNA clusters is the formation of a hybrid lineage or a homoploid hybrid speciation (hybridization without a change in chromosome number, Arnold 1996). Most likely this scenario was realized in *Pinus* (Pinaceae) and freshwater fishes (Cyprinidae) homoploid hybrids (Liu et al. 2003, Pereira et al. 2013). In the case of *Pinus*, *Polyommatus densata* has nine major rDNA clusters in haploid karyotype as a combination of rDNA clusters inherited from the paternal genomes. Similarly, homoploid cyprinid hybrids have rDNA patterns within the range of possible combinations of parental contributions.

On the basis of rDNA evolutionary dynamics and the repetitive structure of rDNA in Lepidoptera Nguyen et al. (2010) proposed ectopic recombination as a possible mechanism of rDNA repatterning. According to this mechanism, non-allelic homologous recombination may take place between homologous rDNA loci located on non-homologous chromosomes. Species with more than one rDNA cluster in combination with an ancestral chromosome number state n=30-31 (*Colias hyale* (Linnaeus, 1758) and *Inachis io* (Linnaeus, 1758) as described in Nguyen et al. 2010) are likely to show evidence for recombination leading to rDNA cluster rearrangements. Thus, karyotype reorganizations which affect the number of rDNA-bearing chromosomes can occur without changes in chromosome number and be a result of ectopic recombination. To conclude, karyotype reorganizations which affect the number of rDNA-bearing chromosomes may occur by multiple mechanisms: chromosome fissions and fusions, hybrid formation and ectopic recombination.

In our study, FISH with telomeric (TTAGG)*_n_* probe conclusively demonstrate that *Polyommatus* (subgenus *Agrodiaetus*) blue butterflies have classical insect telomere organization. On small chromosomes of Polyommatus (Agrodiaetus) peilei, Polyommatus (Agrodiaetus) karindus and Polyommatus (Agrodiaetus) pfeifferi, originated by fragmentations, telomeric signals are also detected. Generally, fissions lead to breakdown in chromosome structure because after this reorganization the newly originated fragmented chromosomes lack telomeres and their chromosome ends need to be protected from degradation (de Lange 2009). Our data indirectly suggest that in *Polyommatus* (subgenus *Agrodiaetus*) this protection system arises after fragmentations *de novo* on the basis of TTAGG repeats.

Appearance of a new telomere seems to be a highly important event in genome evolution, however its proximate and ultimate mechanisms are still unknown. *Polyommatus* (subgenus *Agrodiaetus*) butterflies with their diverse karyotypes represent a good model system for studying these processes.
